# Investigation of 15q11-q13, 16p11.2 and 22q13 CNVs in Autism Spectrum Disorder Brazilian Individuals with and without Epilepsy

**DOI:** 10.1371/journal.pone.0107705

**Published:** 2014-09-25

**Authors:** Danielle P. Moreira, Karina Griesi-Oliveira, Ana L. Bossolani-Martins, Naila C. V. Lourenço, Vanessa N. O. Takahashi, Kátia M. da Rocha, Eloisa S. Moreira, Estevão Vadasz, Joanna Goes Castro Meira, Debora Bertola, Eoghan O’ Halloran, Tiago R. Magalhães, Agnes C. Fett-Conte, Maria Rita Passos-Bueno

**Affiliations:** 1 Centro de Pesquisas sobre o Genoma Humano e Células Tronco, Departamento de Genética e Biologia Evolutiva, Instituto de Biociências, Universidade de São Paulo, São Paulo, Brasil; 2 Departamento de Biologia Molecular, Faculdade de Medicina de São José do Rio Preto, São José do Rio Preto, SP, Brasil; 3 Instituto de Psiquiatria do Hospital das Clínicas, Faculdade de Medicina, Universidade de São Paulo, São Paulo, Brasil; 4 Instituto da Criança da Faculdade de Medicina, Universidade de São Paulo, São Paulo, Brasil; 5 Academic Centre on Rare Diseases, School of Medicine and Medical Science, University College Dublin, Dublin, Ireland; 6 National Children’s Research Centre, Our Lady’s Children’s Hospital, Dublin, Ireland; Instituto Butantan, Brazil

## Abstract

Copy number variations (CNVs) are an important cause of ASD and those located at 15q11-q13, 16p11.2 and 22q13 have been reported as the most frequent. These CNVs exhibit variable clinical expressivity and those at 15q11-q13 and 16p11.2 also show incomplete penetrance. In the present work, through multiplex ligation-dependent probe amplification (MLPA) analysis of 531 ethnically admixed ASD-affected Brazilian individuals, we found that the combined prevalence of the 15q11-q13, 16p11.2 and 22q13 CNVs is 2.1% (11/531). Parental origin could be determined in 8 of the affected individuals, and revealed that 4 of the CNVs represent de novo events. Based on CNV prediction analysis from genome-wide SNP arrays, the size of those CNVs ranged from 206 kb to 2.27 Mb and those at 15q11-q13 were limited to the 15q13.3 region. In addition, this analysis also revealed 6 additional CNVs in 5 out of 11 affected individuals. Finally, we observed that the combined prevalence of CNVs at 15q13.3 and 22q13 in ASD-affected individuals with epilepsy (6.4%) was higher than that in ASD-affected individuals without epilepsy (1.3%; p<0.014). Therefore, our data show that the prevalence of CNVs at 15q13.3, 16p11.2 and 22q13 in Brazilian ASD-affected individuals is comparable to that estimated for ASD-affected individuals of pure or predominant European ancestry. Also, it suggests that the likelihood of a greater number of positive MLPA results might be found for the 15q13.3 and 22q13 regions by prioritizing ASD-affected individuals with epilepsy.

## Introduction

Autism Spectrum Disorder (ASD) is a complex genetic disorder characterized by impaired social interaction and communication, and restricted, repetitive and stereotyped behavior patterns. ASD affects about 1% of the world population [1]–[3] and it occurs four times more commonly in males than in females [4]. In Brazil, a lower prevalence of ASD (0.27%) has been estimated, which was attributed to misdiagnosis [5]. In addition to the core symptoms, over 60% of the ASD-affected individuals can present other clinical conditions, such as epilepsy (∼30%), gastrointestinal problems (9–70%), attention deficit and hyperactivity disorder – ADHD – (∼30%) and sleep disturbance (∼50%) [6]–[8].

Genome-wide screenings for copy number variations (CNVs) have revealed their occurrence in 10 to 20% of ASD individuals [9]–[12], in which the great majority of CNVs is usually rare and private. Exceptions to this rule are CNVs at 15q11-q13, 16p11.2 and 22q13, which, combined, have been found in roughly 3 to 5% of ASD-affected individuals [10], [13]–[15]. While most CNVs at 16p11.2 are about 600 kb [14], CNV sizes in the other two regions vary widely. The 15q11-q13 region is particularly complex, with five breakpoint clusters (BP1-BP5) that define chromosome segments more prone to genomic rearrangements. Of these, the most recurrent chromosomal abnormality among ASD-affected individuals is a 15q11-q13 duplication (between BP2 and BP3, and about 4.95 Mb) and CNVs at 15q13.2-q13.3 (between BP4 and BP5 and ranging in size from 500kb to 2 Mb) [16]–[19]. The size of the 22q13 CNVs ranges from 100 kb to 9 Mb and always involves *SHANK3* [20], [21].

CNVs at 15q11-q13, 16p11.2 and 22q13 have also been associated with other neurological conditions, such as epilepsy, schizophrenia and ADHD [22]–[26]. Apart from the variable clinical expressivity, these CNVs may exhibit incomplete penetrance [11], [23], [27]. The mechanism underlying the incomplete penetrance and the variable expressivity is not fully understood and it seems to depend on multiple hits [28]. Furthermore, the prevalence of these CNVs in distinct ASD subgroups (for instance, in ASD-affected individuals with epilepsy as compared to those without epilepsy) is unknown. Establishing clinical criteria to increase the likelihood of positive results for these alterations is important to prioritize genetic testing resources.

Whole-genome screening of CNVs in populations around the world have shown that their frequencies vary according to the ethnic background, allowing the distinction of populations of European, African and Asian ancestries [29], [30]. Studies of CNVs at 15q11-q13, 16p11.2 and 22q13 have mostly been conducted in populations of pure or predominant European ancestry. It is not known whether they are also prevalent among ASD-affected individuals in populations of other ancestries, such as the Brazilian population, which is tri-hybrid, with important African and Amerindian contributions in addition to the European ancestry [31].

Thus, we conducted the present study to estimate the combined frequency of CNVs at 15q11-q13, 16p11.2 and 22q13 within a group of 531 Brazilian ASD-affected individuals, and we also sought to determine the frequency of CNVs in those regions by taking into account the epileptic and non-epileptic subgroups. Finally, we aimed at investigating whether the individuals with CNVs at the 15q11–13, 16p11.2 and 22q13 regions harbor additional CNVs, through a genome-wide SNP-array analysis.

## Materials and Methods

### Subjects

This study was approved by the Ethics Committee of the Instituto de Biociencias (IB) – Universidade de Sao Paulo (USP). Written informed consent was obtained from all patients’ caregivers upon receiving information about the study.

Five hundred and thirty one Brazilian ASD-affected individuals were recruited for this study and ascertained at the “Centro de Pesquisa sobre o Genoma Humano e Células Tronco” (CEGH-Cel), IB-USP, following previously standardized criteria [32]–[34], which included a detailed anamnesis – pregnancy history, development history, age at onset, and weight, height and head circumference measurements – and a pedigree analysis. All probands were diagnosed according to Diagnostic and Statistical Manual of Mental Disorders, Fourth Edition (DSM-IV) criteria by psychiatrists from Instituto de Psiquiatria, Hospital das Clinicas - Universidade de Sao Paulo (IPq-USP). Whenever possible, an interview based on Autism Diagnostic Interview-Revised (ADI-R) and Childhood-Autism Rating Scale (CARS) evaluation was applied, as previously reported [32]. Epilepsy diagnosis was based on the occurrence of at least two unprovoked seizure episodes occurring more than 24 hours apart. Whenever possible, additional neurological and laboratorial tests were used to complement the diagnosis.

Blood samples from probands and parents were obtained for genomic DNA isolation, which was performed using the Autopure LS automated workstation, following manufacturer’s procedures (Gentra Systems, Minneapolis, US). All the affected boys tested negative for Fragile X Syndrome [35].

### Multiplex Ligation-dependent Probe Amplification (MLPA) analysis

MLPA probe sets targeting the chromosomal regions 15q11-q13, 16p11.2 and 22q13, SALSA MLPA kits P343-B1 and P343-C1 that contain 52 probes (9 control probes, 3 covering chr22∶51,115,059–51,160,754, 11 covering chr16∶28,997,152–30,365,260, and 29 covering chr15∶25,297,217–32,988,875). They were purchased from MRC-Holland (Amsterdam, Netherlands) and used according to the manufacturer’s protocol. The amplification products were identified and quantified by capillary electrophoresis on an ABI 3730 DNA analyzer (Applied Biosystems, Forster City, CA, US). The data were analyzed using GeneMarker software v1.95 (SoftGenetics, State College, PA, US). Threshold values for the peak height ratio were set at 0.75 and 1.3 for deletions and duplications, respectively.

### Microsatellite genotyping

Seven microsatellite markers spanning the 15q11-q13 region were genotyped to identify the parental origin of the duplication of patient 2. The markers D15S1002, D15S1007 and D15S1012, ABI PRISM Linkage Mapping Set version 2.0 (Applied Biosystems, Forster City, CA, US) were genotyped following the manufacturer’s protocol. The other primer sequence pairs, D15S1043, D15S976, D15S1031 and D15S1010, were obtained from the UCSC human genome browser (http://genome.ucsc.edu/cgi-bin/hgGateway, Feb 2009 GRCh37/hg19) and a M13 tail was added to the 5′-end of each forward primer [36]. Microsatellite genotyping was performed by means of an ABI DNA Analyzer (Applied Biosystems, Forster City, CA, US). Analysis of the results was performed with the GeneMarker v1.95 software (SoftGenetics, State College, PA, US).

### CNV prediction analysis from genome-wide SNP arrays

CNV prediction analysis from genome-wide SNP arrays was carried out using the Affymetrix platform (Affymetrix, Santa Clara, CA, US): GeneChip Human Mapping 100K for ASD-affected individuals 10, as described in [37], and 11, GeneChip Human Mapping 500K Array Set for families of ASD-affected individuals 2 and 3, and Genome-Wide Human SNP Array 6.0 for the remaining ASD-affected individuals and parents. Protocols were performed according to the manufacturer’s recommendations.

Data analyses were carried out with Affymetrix Genotyping Console (Affymetrix, Santa Clara, CA, US) and PennCNV (http://www.openbioinformatics.org/penncnv/), using the hg19 assembly (Genome Reference Consortium GRCh37). In both analyses we used the default parameters recommended by the manufacturers. A deletion or duplication was considered for further analyses only when detected by both methods.

We considered a CNV as potentially pathogenic according to two criteria: 1) they contained genes previously associated with ASD and/or other neuropsychiatric and neurological disorders (e.g. ADHD, global developmental delay, intellectual disability, schizophrenia and epilepsy/seizure); and/or 2) they exhibited a minimum overlap of 50% in length with CNVs previously associated with these conditions. For this, we searched Simons Foundation Autism Research Initiative (SFARI – https://gene.sfari.org/autdb/Welcome.do) and Decipher (https://decipher.sanger.ac.uk/) databases. Even though we analyzed, whenever possible, if the CNVs had been inherited, we did not include this information in the classification criteria. The CNVs considered as potentially pathogenic were not found or occurred with a frequency <1% in the Database of Genomic Variants (DGV - http://dgv.tcag.ca/dgv/app/home) [38], [39].

Genome-wide SNP-array data are available in the ArrayExpress database (www.ebi.ac.uk/arrayexpress) under accession numbers E-MTAB-2818, E-MTAB-2819, E-MTAB-2820, E-MTAB-2821 and E-MTAB-2823.

### Ancestry Analysis

We analyzed the ancestry of 9 out of 11 Brazilian ASD-affected individuals in whom high density genotyping (GeneChip Human Mapping 500K Array Set and Genome-Wide Human SNP Array 6.0) was carried out. The PLINK tool set (http://pngu.mgh.harvard.edu/purcell/plink/) [40] was used to merge the Brazilian dataset with the Human Genome Diversity Project (HGDP) [41] and HapMap project [42] datasets, and to select SNPs with a missing call inferior to 1% (geno option to 0.01), which yielded 84,805 SNPs. Next, we used Ancestry Mapper package from R, to produce AMids [43]. Admixture was used to produce ancestral proportions for each individual [44]. The R statistical language and environment [45] was used in most of the analysis, including the visualization, plotting data and clustering algorithms. Python was used to parse data and in some of the analysis.

### Statistical Analysis

To assess the differences in CNV prevalence between the two subgroups, ASD-affected individuals without (N = 453) and with epilepsy (N = 78), we conducted two-tailed Fisher’s exact tests. P-values below 0.05 were considered statistically significant.

## Results

**Figure 1:**
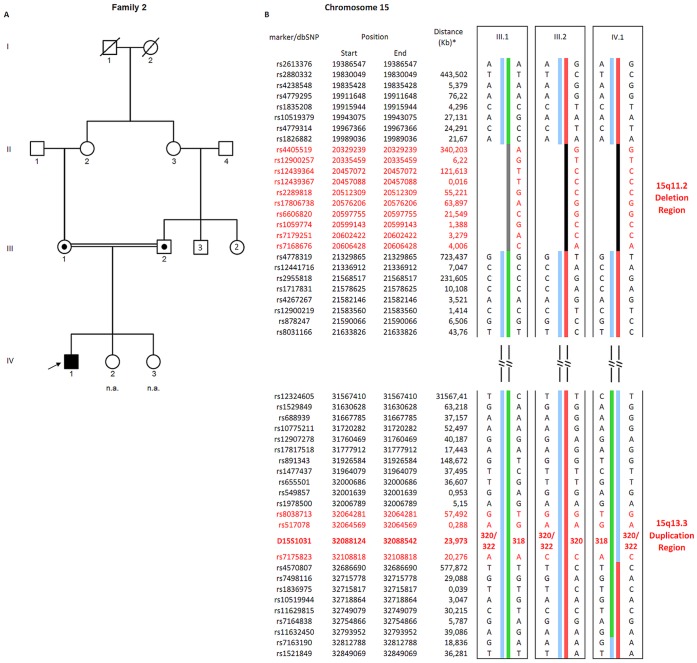
ASD-affected individual 2 pedigree and haplotypes. A) Pedigree of the ASD-affected individual. B) Haplotype analysis of SNPs and microsatellite markers at 15q11-q13. ASD-affected individual – filled symbol; Carriers of 15q13.3 duplication and 15q11.2 deletion – symbols with a black dot in the middle; individuals unavailable or not affected – empty symbols; non-available individuals – n.a. * Distance (in kilobases) from anterior to posterior marker/dbSNP. The other microsatellite markers are not showed.

Through MLPA analysis, we identified CNVs at 15q11-q13 between BP4 and BP5 (15q13.3), and at 16p11.2 and 22q13, respectively, in three (0.6%), five (0.9%), and three (0.6%) of the 531 (423 boys and 108 girls) Brazilian ASD–affected individuals (Table 1; clinical characteristics in Table S1), which are ethnically admixed (Table S2), with a combined prevalence of 2.1% (11/531). In four of eight of those individuals, whose parents were available for genetic testing, the CNVs were found to be *de novo* (affected individuals 1, 6, 10 and 11). Among the other four individuals (affected individuals 2, 3, 4 and 7), only one CNV was maternally inherited (affected individual 7) (Table 1). The parents of affected individual 2 are consanguineous and both are carriers of the CNV at 15q13.3. The parents share a haplotype at this region, suggesting a common origin of the 15q13.3 duplication. Therefore, they probably inherited the CNV from their mothers, who are sisters, while the proband inherited it from his father (Figure 1A and 1B). None of the carrier parents reported behavioral or neurological issues.

**Table 1:** CNVs at the chromosomal regions 15q13.3, 16p11.2 and 22q13 in the Brazilian individuals with ASD.

		MLPA detected CNVs					CNVs detected by SNP-array						
AffectedIndividual	Region	Position (hg19)	Type	Size(Mb)	N Genes	Inheritance	Region	Position (hg19)	Type	Size (Mb)	Inheritance	Disease association	
												Neurol. Dis.	ASD
1	15q13.2-q13.3	chr15∶30,941,572–32,509,926	del	1.56	8	*de novo*	2q13	chr2∶110,453,976–111,084,885	dup	0.631	pat	+	+
2*	15q13.3	chr15∶32,024,192–32,509,926	dup	0,486	1	pat	15q11.2	chr15∶22,410,242–23,222,284	del	0.812	mat	+	+
3	15q13.3	chr15∶31,956,036–32,511,581	dup	0,555	1	pat	–	–	–		–		
4	16p11.2	chr16∶29,696,973–30,191,907	dup	0,495	25	Pat	4q35.2	chr4∶186,934,286–187,137,146	dup	0.203	mat	+	+
							11p11.2	chr11∶48,380,903–48,968,027	del	0.587	pat	+	+
5	16p11.2	chr16∶29,517,699–30,191,895	dup	0,674	28	father n.a.	–	–	–	–	–		
6	16p11.2	chr16∶29,613,495–30,190,030	dup	0,576	27	*de novo*	–	–	–	–	–		
7	16p11.2	chr16∶29,402,301–30,226,931	dup	0,824	32	Mat	–	–	–	–	–		
8	16p11.2	chr16∶29,517,699–30,191,895	del	0.674	28	father n.a.	7p11.2	chr7∶57,260,919–57,882,330	dup	0.621	father n.a.	+	+
9	22q13.3	chr22∶51,027,581–51,234,443	del	0.206	6	father n.a.	17q11.2	chr17∶25,974,257–26,075,524	del	0.101	father n.a.	–	+
10¥	22q13.3	chr22∶50,282,986–51,304,566	del	1.02	35	*de novo*	–	–	–	–	–	–	–
11	22q13.3	chr22∶49,033,233–51,193,680	del	2.27	37	*de novo*	–	–	–	–	–	–	–

**Neurol. Dis.** - Neurological Disorder; “**–**” – not reported in the literature; “**+**” reported in the literature; del – deletion; dup – duplication; n.a. – not available; pat - paternal; mat - maternal;

¥ - ASD-affected individual 10 was described in [37]; * CNVs at 15q13.3 and 15q11.2 are present in both parents;

We conducted a CNV prediction analysis from genome-wide SNP arrays in the 11 ASD-affected individuals in whom we detected CNVs at 15q13.3, 16p11.2 and 22q13 regions to determine their sizes as well as to verify whether another potentially pathogenic CNV would be present. The 15q13.3, 16p11.2 and 22q13 CNVs sizes ranged from 206 kb to 2.27 Mb (Table 1). It is worth noting that the two 15q13.3 duplications were about 500 kb and included only the gene *CHRNA7*. Six additional CNVs (3 duplications and 3 deletions) were identified in 5 of those affected individuals (1, 2, 4, 8 and 9), two of the CNVs in individual 4 (Table 1). Four of these CNVs were inherited, while the parents of the other two affected individuals were unavailable for testing (Table 1). The 15q11.2 CNV in affected individual 2 was also present in both consanguineous parents and the maternal copy was transmitted to the affected proband (Figure 1B). Ancestry analysis was conducted in 9 out of 11 ASD-affected individuals (Table S2), which showed that they have the three main ancestral components commonly observed in the Brazilian population.

Next, we evaluated if CNVs at 15q13.3, 16p11.2 and 22q13 occurred more often among ASD-affected individuals with epilepsy. In our total sample, 78 (54 boys and 24 girls) of the 531 ASD-affected individuals had history of epilepsy (Table 2). We observed that 6 of the 453 ASD-affected individuals without epilepsy (1.3%) and 5 of the 78 ASD-affected individuals with epilepsy (6.4%) had CNVs in one of these regions. These frequencies were significantly different (p = 0.014; odds ratio = 5.1; 95% CI 1.19–20.55). CNVs at 15q13.3 and 22q13 among ASD-affected individuals with epilepsy were responsible for these differences (Table 2).

**Table 2:** Main findings in ASD Brazilian individuals with and without epilepsy.

	Total (N = 531)	ASD without epilepsy (N = 453)	ASD with epilepsy (N = 78)
**Gender**			
* Male*	423	369	54
* Female*	108	84	24
* Ratio (m:f)*	4∶1	4.4∶1	2.3∶1
**Mean age, years (mean ± sd)**	10.2±6.6	13.2±6.5	9.7±6.5
**CNVs at 15q13.3, 16p11.2 and 22q13 (%)**	11 (2.1)	6 (1.3)	5 (6.4) *
**CNVs at 15q13.3, 22q13 (%)**	6 (1.1)	1 (0.02)	5 (6.4)

m – male; f – female; sd, standard deviation; * OR = 5.1; (p-value = 0.014).

## Discussion

In the Brazilian sample of ASD-affected patients, the frequencies of CNVs at 15q13.3 (0.6%), 16p11.2 (0.9%), and 22q13 (0.6%) are consistent with those described in the literature [28], [38]–[42]. The Brazilian population is ethnically admixed, with around 56–62% of European contribution [31], [46]–[48]. In ASD-affected individuals positive for CNVs at 15q11-q13, 16p11.2 and 22q13, we observed that the contribution of European ancestry varied from 38 to 98%. This preliminary analysis suggests that the ethnic admixture of our population is not influencing the occurrence of these CNVs.

We identified both inherited and *de novo* CNVs at 15q11-q13 and 16p11.2, as previously described by other groups [49]–[52]. Indeed, it has been established that maternally and paternally inherited as well as *de novo* 16p11.2 microdeletions and microduplications contribute to the ASD phenotype [51], [53]–[55]. Additionally, 15q13.3 deletions and maternally inherited duplications at 15q11-q13 have been implicated in an increased risk of ASD [28], [56], [57]. However, the association between the 15q13.3 duplication, involving only *CHRNA7,* and neuropsychiatric disorders is still controversial [24], [49], [58]–[60]. In our sample, the two 15q13.3 duplications were inherited from the father, but both maternally and paternally inherited 15q13.3 duplications have been associated with the ASD phenotype [49], [58]. Finally, our data on the origin of the 22q13 deletions provide additional support to the hypothesis that they usually represent *de novo* mutations [21], [61], [62].

The size of the CNVs at 16p11.2 (600kb) and 15q13.3 (500kb or 1.6Mb), are within the range of those previously reported [49], [54], [57], as expected due to the presence of segmental duplications that flank these regions and mediate rearrangements through non-allelic homologous recombination [49], [54], [63]. At 22q13 the size of the CNVs were quite variable, but always included *SHANK3.* Loss-of-function mutations involving *SHANK3* cause Phelan-McDermid syndrome, an autosomal dominant condition with full penetrance that presents ASD among other clinical features [21], [64]. In addition to the aforementioned CNVs, five ASD-affected individuals also carried at least another CNV with no correlation with the presence of CNVs at 15q13.3, 16p11.2 or 22q13. All those additional CNVs detected by the SNP-array platform overlap partially or completely CNVs previously associated with ASD or other neurological conditions [15], [65]–[74]. Therefore, these additional CNVs, which were found in nearly 50% of our affected individuals, might contribute to the penetrance of the ASD phenotype, in accordance with the two- or multiple hit hypotheses for ASD, that is, these CNVs are not the cause ASD alone and depend on the presence of at least a second mutation [58], [75]. Further studies will be necessary for testing their effect and specificity on the phenotype.

Our data suggest that CNVs at 15q13.3 and 22q13 are more prevalent among ASD-affected individuals with epilepsy than among those only with ASD. Indeed, it has been shown that 15q13.3 and 22q13 deletions represent strong genetic risk factors for ASD and epilepsy [76]–[79]. However, the contribution of 15q13.3 duplications, particularly those encompassing only *CHRNA7*, to both phenotypes is still uncertain [19], [49], [58]. Although the 15q13.3 duplication has been implicated in several psychiatric conditions [24], [60], [80], rare cases presented epilepsy as well [49], [81], [82]. Within this context, ASD-affected individual 2 deserves special attention: while his 15q13.3 duplication was paternally inherited, his parents, who do not have any history of epilepsy, probably inherited the duplication from their mothers (who are sibs). This individual also harbors a deletion at 15q11.2 (Table 1), which in turn was inherited from his mother, even though both parents carry this 15q11.2 deletion (Figure 1). *De novo* or inherited deletions at 15q11.2 have been associated with both epilepsy and ASD, however the relative risk that this CNV confers to ASD is low [70], [83], [84]. It is thus possible that CNVs both at 15q11.2 and 15q13.3 are causative factors of ASD and/or epilepsy, supporting the etiologic models that involve multiple genetic alterations in ASD, since both alterations have incomplete penetrance but have already been reported in ASD and other neuropsychiatric diseases.

None of our ASD individuals with epilepsy carry a 16p11.2 CNV. Even though CNVs at 16p11.2 have been associated with epilepsy, this finding is not unexpected, as the phenotype of patients with these CNVs is extremely variable and the overlap between ASD and epilepsy is not often observed among them [55], [85]–[88].

In summary, this work describes the combined prevalence of CNVs at 15q13.3, 16p11.2 and 22q13 as 2.1% in Brazilian ASD-affected individuals. CNVs at 15q13.3 and 22q13 were significantly more frequent in ASD-affected individuals with epilepsy in our sample; hence, these CNVs should be preferentially screened in ASD-affected individuals if resources are limited. Other potentially pathogenic CNVs were identified in 5 out of 11 ASD-affected individuals studied, thus highlighting the need for understanding how those and other genetic alterations interact to give rise to ASD and other clinical complications.

## Supporting Information

Table S1 **Phenotypic characteristics of ASD individuals with CNVs at the 15q13.3, 16p11.2 and 22q13 regions.**
(XLS)

Table S2 **Ancestral Contributions in Brazilian ASD-affected individuals.**
(XLSX)
